# COVID-DeepPredictor: Recurrent Neural Network to Predict SARS-CoV-2 and Other Pathogenic Viruses

**DOI:** 10.3389/fgene.2021.569120

**Published:** 2021-02-11

**Authors:** Indrajit Saha, Nimisha Ghosh, Debasree Maity, Arjit Seal, Dariusz Plewczynski

**Affiliations:** ^1^Department of Computer Science and Engineering, National Institute of Technical Teachers' Training and Research, Kolkata, India; ^2^Department of Computer Science and Information Technology, Institute of Technical Education and Research, Siksha ‘O’ Anusandhan (Deemed to Be University), Bhubaneswar, India; ^3^Department of Electronics and Communication Engineering, MCKV Institute of Engineering, Howrah, India; ^4^Cognizant Technology Solutions Pvt. Ltd., Kolkata, India; ^5^Laboratory of Bioinformatics and Computational Genomics, Faculty of Mathematics and Information Science, Warsaw University of Technology, Warsaw, Poland; ^6^Laboratory of Functional and Structural Genomics, Centre of New Technologies, University of Warsaw, Warsaw, Poland

**Keywords:** long-short term memory, SARS-CoV-2, sequence analysis, virus prediction, genomic information

## Abstract

The COVID-19 disease for Novel coronavirus (SARS-CoV-2) has turned out to be a global pandemic. The high transmission rate of this pathogenic virus demands an early prediction and proper identification for the subsequent treatment. However, polymorphic nature of this virus allows it to adapt and sustain in different kinds of environment which makes it difficult to predict. On the other hand, there are other pathogens like SARS-CoV-1, MERS-CoV, Ebola, Dengue, and Influenza as well, so that a predictor is highly required to distinguish them with the use of their genomic information. To mitigate this problem, in this work COVID-DeepPredictor is proposed on the framework of deep learning to identify an unknown sequence of these pathogens. COVID-DeepPredictor uses Long Short Term Memory as Recurrent Neural Network for the underlying prediction with an alignment-free technique. In this regard, *k*-mer technique is applied to create Bag-of-Descriptors (BoDs) in order to generate Bag-of-Unique-Descriptors (BoUDs) as vocabulary and subsequently embedded representation is prepared for the given virus sequences. This predictor is not only validated for the dataset using K-fold cross-validation but also for unseen test datasets of SARS-CoV-2 sequences and sequences from other viruses as well. To verify the efficacy of COVID-DeepPredictor, it has been compared with other state-of-the-art prediction techniques based on Linear Discriminant Analysis, Random Forests, and Gradient Boosting Method. COVID-DeepPredictor achieves 100% prediction accuracy on validation dataset while on test datasets, the accuracy ranges from 99.51 to 99.94%. It shows superior results over other prediction techniques as well. In addition to this, accuracy and runtime of COVID-DeepPredictor are considered simultaneously to determine the value of *k* in *k*-mer, a comparative study among *k* values in *k*-mer, Bag-of-Descriptors (BoDs), and Bag-of-Unique-Descriptors (BoUDs) and a comparison between COVID-DeepPredictor and Nucleotide BLAST have also been performed. The code, training, and test datasets used for COVID-DeepPredictor are available at *http://www.nitttrkol.ac.in/indrajit/projects/COVID-DeepPredictor/*.

## 1. Introduction

The first case of COVID-19 surfaced in Wuhan, China in December 2019 (Huang et al., 2020; Meng et al., 2020; Yan L. et al., 2020). In no time it spread to 212 countries and territories (Worldometer, 2021) worldwide creating a pandemic in its wake. SARS-CoV-2 falls in the same family as SARS-CoV and MERS-CoV (all belong to the family of coronavirus) and mainly targets the respiratory system (Zhou et al., 2020). As of 8th January 2021, over 885 million cases of COVID-19 have been reported worldwide, with more than 1,906 thousand cases of death and 63.6 million cases of recovery (Worldometer, 2021).

SARS-CoV-2 is defined as an enveloped, positive-sense, single-stranded RNA virus with a genome of around 30 kilobases in length (Weiss and Navas-Martin, 2005; Su et al., 2016; Cui et al., 2019). RNA viruses generally have very high mutation rates (Jenkins et al., 2002; Woo et al., 2009). Genetic mutation can occur infrequently between viruses of the same species but of divergent lineages. The resulting mutated viruses may sometimes cause an outbreak of infection in humans e.g., the case of SARS-CoV-2. Coronavirus results from zoonotic transmission to human and shows symptoms of pneumonia, fever, and breathing difficulties (Guan et al., 2003; Alagaili et al., 2014). Human to human transmission has also been confirmed for SARS-CoV-2 (Chan et al., 2020; Huang et al., 2020). Next-generation sequencing using metagenomic analysis has recently been used to identify the genetic features of SARS-CoV-2 (Zhou et al., 2020).

There have been several analysis regarding SARS-CoV-2. This include whole genome analysis of a virus and viral protein-based comparisons which have resulted in the conclusion that SARS-CoV-2 is mostly related to two bat SARS-like coronaviruses (Chan et al., 2020; Lu et al., 2020). Phylogenetic analysis of full genome alignment and similarity plot show that SARS-CoV-2 has high similarity with bat coronavirus *RaTG13* (Paraskevis et al., 2020). Furthermore, another study (Wan et al., 2020) has shown that spike protein receptor-binding domain (RBD) of SARS-CoV-2 binds with host receptor angiotensin-converting enzyme 2 (ACE2), just like other *Sarbecovirus* strains, thus making the claim that SARS-CoV-2 originated from bat very likely (Letko et al., 2020; Liu and Wang, 2020).

As the genomic structure of SARS-CoV-2 is similar to the other viruses of the same family, and it shows similar symptoms like them, the early prediction of SARS-CoV-2 is a very challenging task. Ozturk et al. (2020) have used deep neural networks with X-ray images for automated detection of SARS-CoV-2 cases. The results show that the method has a prediction accuracy of 98.08% for binary classes (COVID vs. No-Findings) and 87.02% for multiple classes (COVID vs. No-Findings vs. Pneumonia). Another work (Yan Q. et al., 2020) where deep learning has been used to predict age-related macular degeneration (AMD) which is a leading cause of blindness among the elderly population. The results show an average area under the curve (AUC) value of 0.85. On the other hand, the authors in Koohi-Moghadam et al. (2019) have used deep learning approach to predict disease-associated mutation of metal-binding sites in proteins. The prediction results depict AUC as 0.90 and an accuracy of 0.82. These encouraging results show that deep learning has the potential for highly accurate prediction. This led us to devise a predictor based on deep learning which uses genomic sequences of pathogenic viruses. In this work, a deep learning technique, viz. COVID-DeepPredictor based on Long-Short Term Memory (LSTM) (Hochreiter and Schmidhuber, 1997; Tang et al., 2019) is developed. Though, LSTM has been profusely used in many works for text classification (Jin et al., 2019; Liu et al., 2019; Zhang et al., 2019), to the best of the authors' knowledge, this is the first attempt to use LSTM for the prediction of SARS-CoV-2 using genomic sequences of virus considering alignment-free approach. For this purpose, *k*-mer technique is used to generate Bag-of-Descriptors (BoDs) and consequently Bag-of-Unique-Descriptors (BoUDs) as vocabulary. Subsequently embedded representation is prepared for the given virus sequences using BoDs and BoUDs. It is worth mentioning that, though SARS-CoV-2 is a single-stranded RNA virus, the genomic information of a virus is captured in the form of DNA sequence. These DNA sequences are used in this work to predict SARS-CoV-2 and other pathogenic viruses viz. SARS-CoV-1, MERS-CoV, Ebola, Dengue, and Influenza. COVID-DeepPredictor achieves 100% prediction accuracy on validation dataset while on test datasets, the accuracy ranges from 99.51 to 99.94%. COVID-DeepPredictor also shows superior results over the existing prediction techniques based on Linear Discriminant Analysis, Random Forests, and Gradient Boosting Method. Moreover, apart from prediction accuracy, critical analysis like the choice of *k* in *k*-mer by considering the accuracy and runtime of COVID-DeepPredictor simultaneously, a comparative study of Bag-of-Descriptors (BoDs) and Bag-of-Unique-Descriptors (BoUDs) for different values of *k* and a comparison between an alignment-based technique viz. Nucleotide Basic Local Alignment Search Tool (BLASTN) and COVID-DeepPredictor as alignment-free technique.

## 2. Materials and Methods

In this section, description of dataset preparation that has been used in this work are elucidated, a brief description of Long-Short Term Memory (LSTM) and the detailed discussion of proposed COVID-DeepPredictor are put forth.

### 2.1. Data Preparation

The datasets of SARS-CoV-1, MERS-CoV, Ebola, Dengue, and Influenza have been downloaded from NCBI (National Center for Biotechnology Information)^1^. Dataset for SARS-CoV-2 has been downloaded from NCBI and GISAID (Global Initiative on Sharing All Influenza Data)^2^. The total number of complete or near-complete genomic sequences of all the pathogenic viruses amounted to 4,643, named as Initial dataset. Additionally, the recent complete or near-complete SARS-CoV-2 sequences of 3,030 during January 2020 to August 2020 are taken from NCBI whereas 2,410 (from February 2020 to July 2020) and 4,000 (from June 2020 to December 2020) sequences are considered from GISAID. For our training purpose, 1,500 samples from 4,643 sequences are taken randomly for training. To ensure that representatives from all the six pathogenic viruses are available and to avoid imbalance class problem, 250 samples from each pathogenic viruses are taken in the training dataset. In order to perform testing, five different test datasets are created and named as Testdata-1, Testdata-2, Testdata-3, Testdata-4, and Testdata-5. It is important to mention that Testdata-1 consists of the remaining 3,143 sequences out of 4,643 sequences, while Testdata-2 contains 200 sequences each for MERS-CoV, SARS-CoV-2, Ebola, Dengue, and Influenza and 90 sequences of SARS-CoV-1 from different sources. Moreover, Testdata-3, Testdata-4, and Tetsdata-5 comprise of recent SARS-CoV-2 sequences from NCBI and GISAID respectively along with other pathogenic viruses. The statistics of Initial dataset as well as training and testing datasets are given in Table 1. It is worth mentioning that in this work more than 10,000 SARS-CoV-2 genomic sequences have been used from January 2020 to December 2020 considering different sources in order to develop COVID-DeepPredictor.

**Table 1:** Description of initial, training, and test datasets.

**Dataset**	**Virus name**	**Number of sequences**	**Max. length of sequence**	**Avg. lengthof sequence**	**Source of sequence**
Initial dataset	SARS-CoV-1	340	30,311	29,515	NCBI-SARS-CoV-1
	MERS-CoV	291	30,150	29,983	NCBI-MERS-CoV
	SARS-CoV-2	2,402	29,986	29,507	GISAID-SARS-CoV-2
	Ebola	300	19,897	18,976	NCBI-Ebola
	Dengue	300	11,195	10,746	NCBI-Dengue
	Influenza	1,010	2,347	2,322	NCBI-Influenza
Training dataset	SARS-CoV-1	250	29,765	29,520	NCBI-SARS-CoV-1
	MERS-CoV	250	30,123	29,999	NCBI-MERS-CoV
	SARS-CoV-2	250	29,927	29,334	GISAID-SARS-CoV-2
	Ebola	250	19,897	18,979	NCBI-Ebola
	Dengue	250	11,195	10,748	NCBI-Dengue
	Influenza	250	2,347	2,333	NCBI-Influenza
Testdata-1	SARS-CoV-1	90	30,311	29,494	NCBI-SARS-CoV-1
	MERS-CoV	41	30,150	29,887	NCBI-MERS-CoV
	SARS-CoV-2	2,152	29,986	29,527	GISAID-SARS-CoV-2
	Ebola	50	19,034	18,964	NCBI-Ebola
	Dengue	50	10,764	10,737	NCBI-Dengue
	Influenza	760	2,341	2,318	NCBI-Influenza
Testdata-2	SARS-CoV-1	90	30311	29494	NCBI-SARS-CoV-1
	MERS-CoV	200	30,423	29,066	NCBI-MERS-CoV
	SARS-CoV-2	200	29,855	29,850	GISAID-SARS-CoV-2
	Ebola	200	18,798	18,762	NCBI-Ebola
	Dengue	200	10,731	10,692	NCBI-Dengue
	Influenza	200	2,341	2,323	NCBI-Influenza
Testdata-3	SARS-CoV-1	90	30,311	29,494	NCBI-SARS-CoV-1
	MERS-CoV	220	30,423	29,162	NCBI-MERS-CoV
	SARS-CoV-2	3,030	29,903	29,780	NCBI-SARS-CoV-2
	Ebola	220	18,871	18,850	NCBI-Ebola
	Dengue	220	10,690	10,677	NCBI-Dengue
	Influenza	220	2,341	2,323	NCBI-Influenza
Testdata-4	SARS-CoV-1	90	30,311	29,494	NCBI-SARS-CoV-1
	MERS-CoV	250	30,423	29,277	NCBI-MERS-CoV
	SARS-CoV-2	2,410	30,423	29,726	GISAID-SARS-CoV-2
	Ebola	250	18,871	18,852	NCBI-Ebola
	Dengue	250	10,757	10,538	NCBI-Dengue
	Influenza	250	2,316	2,316	NCBI-Influenza
Testdata-5	SARS-CoV-1	90	30,311	29,494	NCBI-SARS-CoV-1
	MERS-CoV	250	30,423	29,277	NCBI-MERS-CoV
	SARS-CoV-2	4,000	29,903	29,798	GISAID-SARS-CoV-2
	Ebola	200	18,798	18,762	NCBI-Ebola
	Dengue	220	10,690	10,677	NCBI-Dengue
	Influenza	250	2,316	2,316	NCBI-Influenza

**Figure 1:**
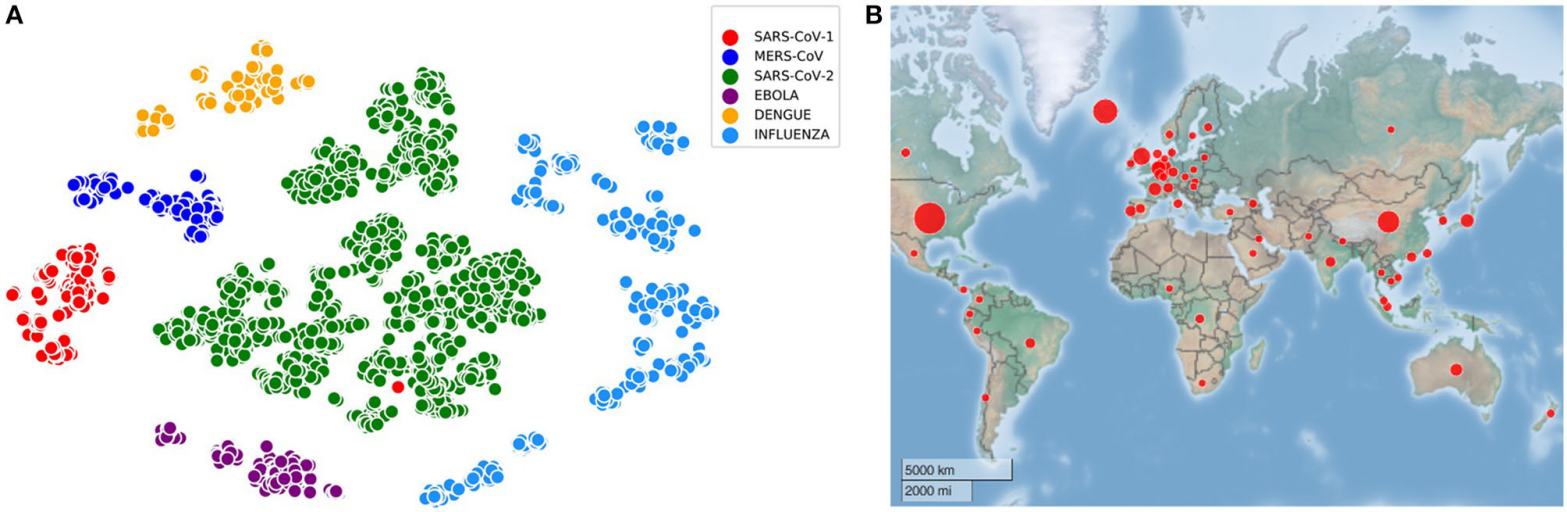
Visualization of virus sequences and their spread. **(A)** Embedded representation of SARS-CoV-1, MERS-CoV, SARS-CoV-2, Ebola, Dengue, and Influenza (*k*=3) **(B)** 56 countries considered for COVID-DeepPredictor are spread across the globe.

All the experiments are performed with the training and testing datasets as mentioned in Table 1. For the visualization of all the virus sequences (SARS-CoV-1, MERS-CoV, SARS-CoV-2, Ebola, Dengue, and Influenza), t-distributed Stochastic Neighbor Embedding (tSNE) (Hinton and Roweis, 2003) is applied on 4,643 sequences after generating the count vector (Khattak et al., 2019) using *k*-mer technique (Manekar and Sathe, 2018; Solis-Reyes et al., 2018). In this regard, the number of clusters known apriori is six and such embedded representation of virus sequences is shown in Figure 1A along with the distribution of initial SARS-CoV-2 sequences in 56 countries in Figure 1B. It is to be noted that COVID-DeepPredictor is developed in MATLAB R2020a.

### 2.2. Long-Short Term Memory

Long-Short Term Memory (LSTM) is a type of recurrent neural network (sub-branch of deep learning) which is capable of learning order dependence in sequence prediction problems. The main components of an LSTM network are sequence input layer and an LSTM layer. A sequence input layer provides text as an input into the LSTM network. An LSTM layer learns long-term association between steps of sequence data. Elaborately speaking, an LSTM network acquires a context vector from previous time step and an input vector from the given data. This is used to calculate the next context and gate vectors to control memory cell state vector (Kim et al., 2018). With an input data at time *t* and a context vector *h*, a raw cell vector and input vectors for each gate are created by one hidden layer. At the input gate, the cell vector is then multiplied by the input vector. The cell input is added to given previous cell vector weighted by the forget vector. Then the resultant vector is controlled by the output vector. The update of the cell is controlled by the control gate. LSTM is mainly trained using Back-propagation Through Time and mitigates the vanishing gradient problem that is quite rampant in RNN. In LSTM, the memory cells and the gates can store time and thus can eliminate old observations overcoming vanishing gradient problem.

To sum up, LSTM consists of four gates, input gate (*i*_*t*_), forget gate (*f*_*t*_), control gate (*C*_*t*_), and output gate (*o*_*t*_). Considering a sentence *S* = *x*_1_, *x*_2_, ..., *x*_*K*_, where *K* is the length of a sentence, the equations for LSTM can be depicted as:

(1) $$\begin{array}{l} i_{t}=sigm\left(W_{i}\times \left[h_{t-1},x_{t}\right]+b_{i}\right) \end{array}$$

(2) $$\begin{array}{l} f_{t}=sigm\left(W_{f}\times \left[h_{t-1},x_{t}\right]+b_{f}\right) \end{array}$$

(3) $$\begin{array}{l} \tilde{C_{t}}=tanh\left(W_{c}\times \left[h_{t-1},x_{t}\right]+b_{c}\right)\\ C_{t}=f_{t}\times C_{t-1}+i_{t}\times \tilde{C_{t}} \end{array}$$

(4) $$\begin{array}{l} o_{t}=sigm\left(W_{o}\times \left[h_{t-1},x_{t}\right]+b_{o}\right)\\ h_{t}=o_{t}*tanh\left(C_{t}\right) \end{array}$$

Here, *W* are weight matrices, *h*_*t*−1_ is the hidden layer which is used updated by the output layer and is also responsible for updating the output and *tanh* and *sigm*, respectively represent the tanh-activation and sigmoid-activation functions.

### 2.3. COVID-DeepPredictor

**Figure 2:**
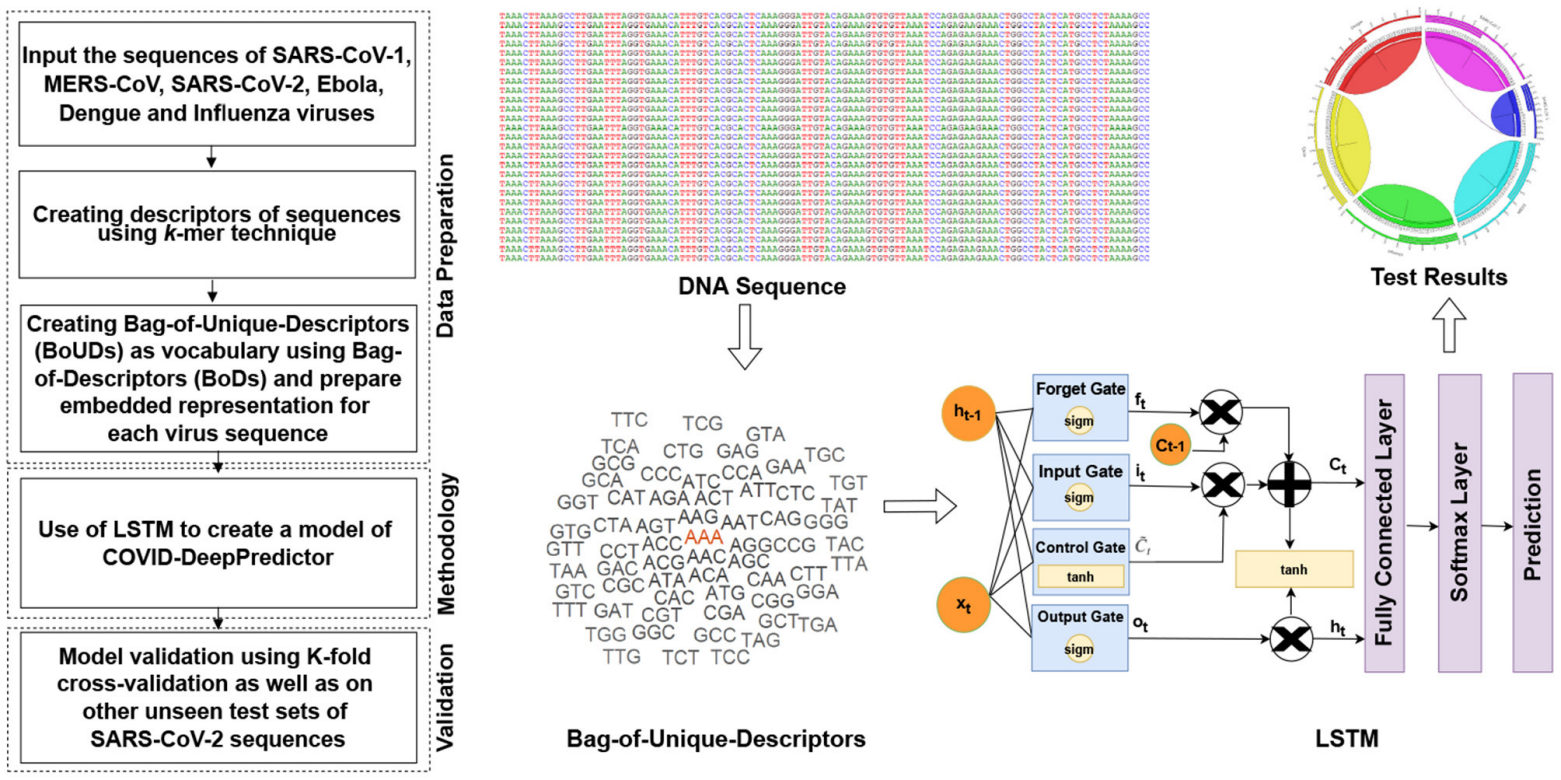
Pipeline of COVID-DeepPredictor. The pipeline is depicted in the form of a flowchart and then represented diagrammatically where the symbols are defined as above.

The main objective of COVID-DeepPredictor is to correctly predict the virus classes based on the given genomic sequences of the different pathogenic viruses using an alignment-free technique. To achieve this, the entire genomic sequence is initially divided into descriptors of sequences called as Bag-of-Descriptors (BoDs) using the popular *k*-mer technique. Here, descriptors are patterns of the genomic sequences of length *k*. Thereafter, Bag-of-Unique-Descriptors (BoUDs) as vocabulary are created using such BoDs. With the use of BoDs and BoUDs, an embedded representation is created of size N × M where N is the number of genomic sequences and M is the indices of the descriptors in vocabulary. This embedded representation is then used to train COVID-DeepPredictor. Since we have divided the genomic sequences into descriptors and represented in the form of tokens, they behave like texts, thus boiling down to a text classification problem. The pipeline of the proposed COVID-DeepPredictor is shown in Figure 2.

## 3. Results

To validate COVID-DeepPredictor, experiments are conducted on genomic sequences of different pathogenic viruses. In this regard, MATLAB R2020a is used on an Intel Core i5-8250U CPU @ 1.80 GHz machine with 8 GB RAM and Windows 10 operating system. The parameters of the underlying predictor, LSTM of COVID-DeepPredictor have been set experimentally. In this regard, the number of hidden units for LSTM layer is set as 80. Next, to use the LSTM layer for a sequence-to-label prediction problem, the output mode is set to “last.” Finally, a fully connected layer with the same size as the number of classes, a softmax layer and a prediction layer are added as well. Mini-batch gradient descent is used to train LSTM. The mini-batch size is specified as 16 and the gradient threshold is set to 2. The COVID-DeepPredictor is compared with other predictors based on Linear Discriminant Analysis (LDA), Random Forests (RFs), and Gradient Boosting Method (GBM). For LDA, the discriminant type is considered to be pseudo-linear, for Random Forests, the number of trees taken are 50 and for GBM the maximum depth of the tree is 10 and maximum iterations are taken as 100. All these parameters are set experimentally.

Each predictor has been evaluated using K-fold cross-validation (K = 10) technique followed by further validation on unseen test datasets. The cross-validation partition uses random non-stratified sampling method which is applied to prepare the training and validation datasets resulting in a total of 1,500 samples. The training and validation datasets consist of all the pathogenic virus classes; SARS-CoV-1, MERS-CoV, SARS-CoV-2, Ebola, Dengue, and Influenza. For each predictor, the descriptors of the sequences of the viruses are created using *k*-mer method. Thereafter to train the COVID-DeepPredictor and the other compared predictors, an embedded matrix of size N × M is created with the use of BoDs and BoUDs.

To determine the performance of COVID-DeepPredictor and the other predictors, *Confusion Matrix* (Luque et al., 2019) is considered. In confusion matrix, **True Positives** (TP) refer to a data being correctly identified and they are represented by the diagonal elements. The remaining predictions lead to an error ϵ. Moreover, **False Positives** (FP) for a particular class refer to the sum of the values in the corresponding column, excluding the TP and **False Negatives** (FN) for a class is the sum of the values in the corresponding row, excluding the TP. Lastly, **True Negatives** (TN) for a class is the sum of all columns and row, barring the one for itself. To evaluate the results of COVID-DeepPredictor, metrics like *Accuracy, Precision, Recall*, and *G-Mean* have been considered which can be deduced from a confusion matrix. They can be calculated as:

***Accuracy***:

(5) $$\begin{array}{l} \frac{TP+TN}{TN+FP+FN+TP} \end{array}$$

***Precision***:

(6) $$\begin{array}{l} \frac{TP}{FP+TP} \end{array}$$

***Recall***:

(7) $$\begin{array}{l} \frac{TP}{TP+FN} \end{array}$$

***G-mean***:

(8) $$\begin{array}{l} \frac{TP}{\sqrt{\left(TP+FP\right)\left(TP+FN\right)}} \end{array}$$

Different existing state-of-the-art predictors based on Linear Discriminant Analysis (LDA), Random Forests (RFs), and Gradient Boosting Method (GBM) are used in this work for comparison purposes. LDA is a very popular machine learning tool for prediction. In LDA, each dependent variable is expressed as a linear combination of other features. RFs are ensemble learning methods which build numerous decision trees during training and as an output produces the class that is the mode of the classes. GBM is also an ensemble learning model which produces a prediction model in the form of an ensemble weak prediction models, usually decision trees.

**Figure 3:**
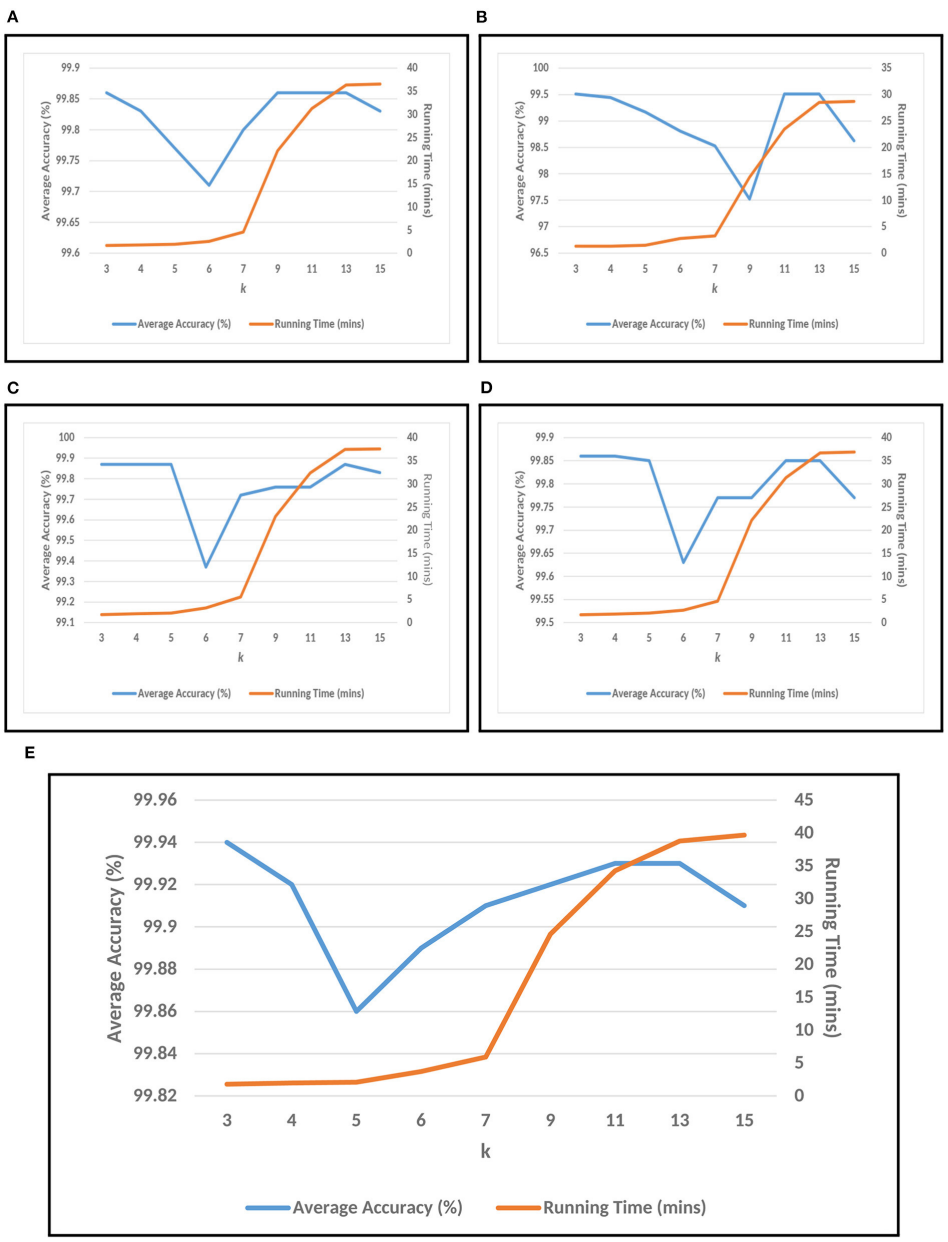
Choosing *k* value of *k*-mer for COVID-DeepPredictor based on accuracy and running time. **(A)** Testdata-1, **(B)** Testdata-2, **(C)** Testdata-3, **(D)** Testdata-4, **(E)** Testdata-5.

**Figure 4:**
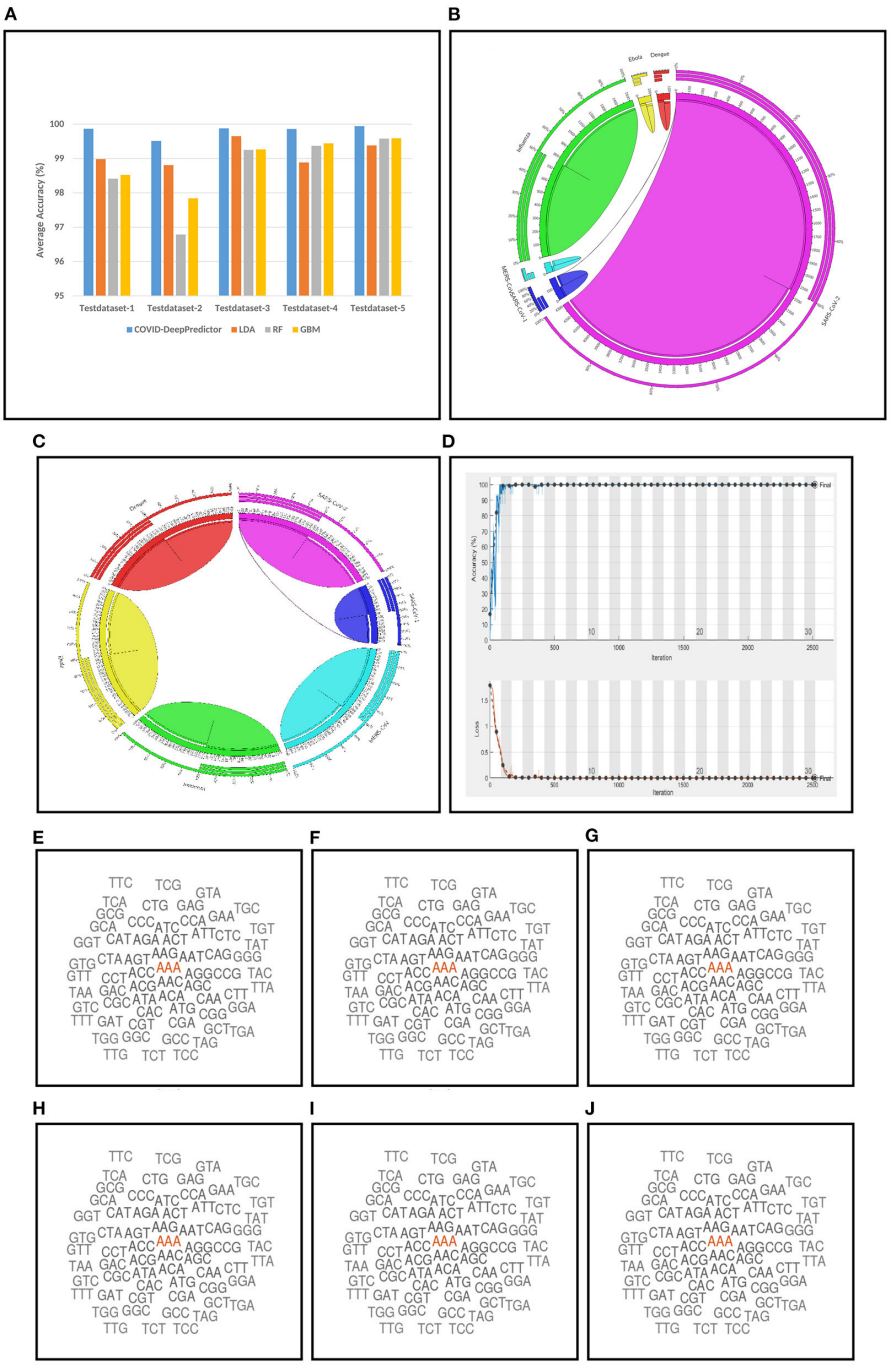
Results related to COVID-DeepPredictor. **(A)** Prediction performance of COVID-DeepPredictor and other compared methods in terms of average accuracy for the five test datasets. Circos plots of confusion matrix for COVID-DeepPredictor (*k*=3) for **(B)** Testdata-1 **(C)** Testdata-2. **(D)** Convergence plot of COVID-DeepPredictor. Word cloud of *k*-mer descriptors (*k*=3) of genome sequences for **(E)** SARS-CoV-1 **(F)** MERS-CoV **(G)** SARS-CoV-2 **(H)** Ebola **(I)** Dengue **(J)** Influenza.

For conducting the experiments, first and foremost, we need to determine the value of *k* in *k*-mer. In order to do this, the experiments have been conducted on five test datasets as mentioned in section 2. The results are shown in Figures 3A–E, where *k* is varied from 3 to 15 with accuracy and running time of COVID-DeepPredictor. It can be seen from figures that the accuracy is higher at *k* = 3 for all the five test datasets. Although, the same accuracy can be found for other *k* values as well, e.g., in Figure 3A *k* = 9, 11, and 13 show the same accuracy, as we increase the *k*-mer value, the run time increases. This trend of increasing time with the increasing value of *k*-mer has also been shown in Solis-Reyes et al. (2018). Keeping this in context, we have taken the value of *k* in *k*-mer to be 3 as with this value, the run time is least. For the compared predictors based on LDA, RF, and GBM, the *k* values are similarly determined as 13, 4, and 4, respectively. In this work, K-fold cross-validation with K = 10 is used. The average results in terms of accuracy for the test datasets are shown in Figure 4A. Moreover, apart from accuracy, different metrics such as precision, recall and g-mean have also been computed for the test datasets and reported in Table 2. As can be seen from the results of Figure 4A, for COVID-DeepPredictor the accuracy ranges from 99.51 to 99.94%. Thus, the experiments establish the fact that COVID-DeepPredictor can detect SARS-CoV-2 with a very high accuracy. The confusion matrices as circos plots for Testdata-1 and Testdata-2 (*k* = 3) are shown in Figures 4B,C. It can be seen from Figures 4B,C that there is only one misprediction, where SARS-CoV-1 has been wrongly predicted as SARS-CoV-2. The confusion matrices for Testdata-3, Testdata-4, and Testdata-5 (*k* = 3) are shown in Supplementary Figure 2.

**Table 2:** Prediction performance of COVID-DeepPredictor and other compared methods on test datasets.

**Method**	**DataSet**	***k*-mer**	**Average accuracy**	**Average precision**	**Average Recall**	**Average G-Mean**
COVID-DeepPredictor	Testdata-1	3	**99.867**	**99.914**	**99.336**	**0.996**
LDA		13	98.981	91.845	98.015	0.948
RF		4	98.409	97.577	90.024	0.937
GBM		4	98.524	97.611	90.121	0.937
COVID-DeepPredictor	Testdata-2	3	**99.513**	**99.527**	**99.423**	**0.994**
LDA		13	98.807	98.814	98.925	0.988
RF		4	96.788	96.981	97.264	0.971
GBM		4	97.844	97.542	97.991	0.977
COVID-DeepPredictor	Testdata-3	3	**99.877**	**99.595**	**99.686**	**0.996**
LDA		13	99.650	98.981	99.162	0.989
RF		4	99.250	97.727	98.440	0.981
GBM		4	99.265	97.728	98.891	0.983
COVID-DeepPredictor	Testdata-4	3	**99.860**	**99.637**	**99.682**	**0.996**
LDA		13	98.885	97.281	97.648	0.974
RF		4	99.371	98.414	99.325	0.988
GBM		4	99.441	98.922	99.444	0.991
COVID-DeepPredictor	Testdata-5	3	**99.940**	**99.766**	**99.808**	**0.997**
LDA		13	99.380	97.467	97.927	0.976
RF		4	99.580	98.519	99.371	0.989
GBM		4	99.590	98.956	99.763	0.993

*The results highlighted in bold show that COVID-DeepPredictor has superior performance as compared to the other predictors*.

COVID-DeepPredictor is performed on a validation dataset as well. Accuracy, precision, recall, and G-mean values of the prediction for the validation dataset are 100, 100, 100, and 1%, respectively (*k*=3). As we have used K-fold cross-validation with K = 10, ten convergence plots of COVID-DeepPredictor are generated. One of the corresponding convergence plots for COVID-DeepPredictor is given in Figure 4D. The blue line indicates the training accuracy and the black line is the validation accuracy. All the convergence plots are shown in Supplementary Figure 1. The Bag-of-Unique-Descriptors of the six virus classes, SARS-CoV-1, MERS-CoV, SARS-CoV-2, Ebola, Dengue, and Influenza are shown in Figures 4E–J for *k*=3.

## 4. Discussion

SARS-CoV-2 is a global pandemic and since human to human transmission (Chan et al., 2020; Huang et al., 2020) is confirmed for SARS-CoV-2, the need for its early prediction has become imperative. Viral outbreaks of this kind call for timely and prompt analysis of the genomic sequences to help the prediction of the virus in its early stages. COVID-DeepPredictor can be used by pathogen laboratories for the prediction of SARS-CoV-2 very quickly and as concluded from the results, most accurately. It is worth mentioning over here that for COVID-DeepPredictor to be effective, there must be at least two virus classes present in the training input sequences.

COVID-DeepPredictor has two functions for: (a) training, testing, and accordingly saving an LSTM model [COVIDdeepPredictor()] and (b) loading a pre-trained LSTM model for testing on unseen test dataset [COVIDdeepPredictorLoad()]. There is a main code COVIDmain.m which loads both COVIDdeepPredictor() and COVIDdeepPredictorLoad(). If users want to have their own training model and also get the results for a test dataset, they need to use only COVIDdeepPredictor() and disable COVIDdeepPredictorLoad(). On the other hand, if they want to use a pre-trained model, they can disable COVIDdeepPredictor() and run only COVIDdeepPredictorLoad() to get the results for test datasets.

For ease of users, training and testing files are provided to make them acquainted with the functionalities of COVIDdeepPredictor(). Trainingdata.csv is the input file for training and any one of the test files among Testdata-1.csv, Testdata-2.csv, Testdata-3.csv, Testdata-4.csv, and Testdata-5.csv can be used for testing. The results of the prediction will have the sequence ID, predicted virus name, along with its sequence which will be stored in Results.csv.

On the other hand, in case of COVIDdeepPredictorLoad(), only any one of the test files needs to be provided to get the results in Results.csv. Similarly, new training and test datasets can be prepared by the users after following the same structures of the training and testing files as provided. This is important so that new training models of COVID-DeepPredictor can be prepared for different set of viruses or similar kind of tasks. It is to be noted that the pre-trained model is provided in Supplementary Material, where the value of *k* for *k*-mer is 3. The choice of *k* has been done experimentally as it takes computationally less amount of time and provides higher accuracy. Sample files for training, testing, pre-trained models for COVID-DeepPredictor and the code of the software are available in Supplementary Material for re-usability^3^.

Setting the appropriate value of *k* in *k*-mer is very important to achieve the desired results in a text classification problem. As this work is based on the underlying concept of text classification, *k*-mer has a very important role to play. Thus, to determine the value of *k* in *k*-mer, extensive experiments have been performed. It can be observed from Figures 3A–E that with the increasing value of *k*, the run time of COVID-DeepPredictor is also on the rise. Therefore, to choose the appropriate value of *k*, apart from the accuracy, the run time of COVID-DeepPredictor also needs to be taken into account. For Testdata-1, at *k* = 9, 11, and 13, the accuracy is same as at *k* = 3. Similarly, for Testdata-2, Testdata-3, Testdata-4, and Testdata-5, similar accuracies can be observed at *k* = 3, 11, 13, *k* = 3, 4, 5, 13, *k* = 3, 4, and *k* = 3, 13, respectively. Although, the accuracies are same at these *k*-mer values, run time is increasing as can been seen from Figures 3A–E. Thus, the smallest *k*-mer value has been chosen without compromising on the accuracy. From Table 2 and Figure 4A, it is quite evident that with *k* = 3, COVID-DeepPredictor shows the best results among all the compared predictors.

To understand the relation among *k*-mer, size of BoDs and BoUDs, Table 3 is reported. From this table, we can see that the sizes of both BoDs and BoUDs increase with the increase in *k*-mer for each virus class. In the table, “All” represents all the six virus classes taken together. For example, at *k* = 15 for training dataset of all virus classes, the sizes of BoDs and BoUDs are 30193594 and 518372, respectively for 1,500 sequences while for the same *k*, for Testdata-1, the sizes of BoDs and BoUDs are 70595908 and 581774 respectively for 3,143 sequences. On the other hand, for *k* = 3, less number of BoDs and BoUDs are generated. Here, as expected, the BoD values for “All” are the summation of the BoDs of the individual virus classes. On the contrary, BoUD is less than the summation of the BoUDs of the six virus classes. This can be attributed to the relatedness between different virus classes. For example, SARS-CoV-1, MERS-CoV, and SARS-CoV-2 are more related and thus they may share unique descriptors (BoUDs) resulting in the intersection of the BoUDs when all the virus classes are considered together. Apart from this, BoDs and BoUDs for the varying *k* have also an impact on the accuracy and run time of COVID-DeepPredictor as well which can be observed by combining Figure 3 and Table 3.

**Table 3:** Bag-of-Descriptors and Bag-of-Unique-Descriptors for each virus class.

***k*-mer**	**Virus Name**	**Training dataset**		**Testdata-1**		**Testdata-2**		**Testdata-3**		**Testdata-4**		**Testdata-5**	
		**BoD**	**BoUD**	**BoD**	**BoUD**	**BoD**	**BoUD**	**BoD**	**BoUD**	**BoD**	**BoUD**	**BoD**	**BoUD**
3	SARS-CoV-1	16000	64	5760	64	5760	64	5760	64	5760	64	5760	64
	MERS	16000	64	2642	81	12831	90	14083	67	16003	67	16003	67
	SARS-CoV-2	16000	64	138336	181	12800	64	193920	64	154240	64	256000	64
	Ebola	16000	64	3200	64	14248	125	14741	125	16661	125	14248	125
	Dengue	16000	64	3212	75	12827	82	14080	64	16496	138	14080	64
	Influenza	16000	64	48688	90	12803	67	14080	64	16000	64	16000	64
	All	96000	64	201838	181	71269	125	256664	125	225160	141	322091	125
5	SARS-CoV-1	255723	1024	92053	1024	92053	1024	92053	1024	92053	1024	92053	1024
	MERS	256000	1024	42012	1054	204674	1081	225101	1029	255821	1029	255821	1029
	SARS-CoV-2	255578	1024	2202055	1446	204592	1023	3099528	1024	2465318	1024	4091752	1024
	Ebola	255966	1024	51195	1024	208766	2461	227294	2104	257985	2104	208766	2461
	Dengue	253210	1024	50659	1044	202616	1054	222741	1024	253923	1493	222741	1024
	Influenza	200176	1022	608293	1093	159407	1020	175272	1015	201513	1007	201513	1007
	All	1476653	1024	3046267	1548	1072108	2555	4041989	2106	3526613	2293	5072646	2452
7	SARS-CoV-1	2804578	15151	1008955	15813	1008955	15813	1008955	15813	1008955	15813	1008955	15813
	MERS	2928952	12897	479752	12526	2293586	15184	2528113	15111	2879852	15113	2879852	15113
	SARS-CoV-2	2649879	12330	22899216	15728	2137492	11100	32349863	14073	25724590	12971	42685988	14211
	Ebola	2443931	13407	490077	14109	1947490	18116	2143135	17557	2435668	17562	1947490	18116
	Dengue	1681474	15764	337983	13206	1332951	15733	1454478	14773	1650576	16470	1454478	14773
	Influenza	513627	10642	1545260	9627	407434	8175	447771	8253	510118	6824	510118	6824
	All	13022441	16365	26761243	17235	9127908	20509	39932315	18815	34209759	19521	50486881	20334
9	SARS-CoV-1	6628103	74045	2384098	99891	2384098	99891	2384098	99891	2384098	99891	2384098	99891
	MERS	6789715	36574	1109206	32462	5266196	68377	5811335	68421	6628997	68503	6628997	68503
	SARS-CoV-2	6477353	39782	56109728	87633	5264550	29600	79603531	62655	63327698	47111	105111057	65922
	Ebola	4441121	38632	888076	42449	3510149	52268	3873871	69072	4403894	69127	3510149	52268
	Dengue	2552607	85437	510925	39245	2032038	84400	2230265	59231	2503617	83849	2230265	59231
	Influenza	576353	25781	1736059	20908	458593	15572	504138	15921	571045	11662	571045	11662
	All	27465252	170456	62738092	176102	18915624	190230	94407238	191127	79819349	194988	120435611	188263
11	SARS-CoV-1	7307627	107764	2628669	164654	2628669	164654	2628669	164654	2628669	164654	2628669	164654
	MERS	7433338	43970	1214507	37565	5761632	93236	6358646	93410	7254330	93587	7254330	93587
	SARS-CoV-2	7255552	50534	62870692	146218	5905735	34664	89280255	94001	71036334	64429	117924347	100857
	Ebola	4708196	47084	940996	50512	3714237	64927	4101614	91849	4663098	91945	3714237	64927
	Dengue	2670007	136386	534074	51172	2126694	135576	508411	19304	2619237	132259	508411	19304
	Influenza	580256	33741	1752556	26635	462340	18759	2334852	85407	576053	13648	576053	13648
	All	29954976	385098	69941518	425910	20599307	465475	105212447	491662	88777721	504060	132606047	448483
13	SARS-CoV-1	7368667	122008	2650637	191450	2650637	191450	2650614	191438	2650614	191438	2650614	191438
	MERS	7491153	47114	1223927	39330	5806329	101342	6408044	101572	7310682	101788	7310682	101788
	SARS-CoV-2	7320339	54634	63432818	171269	5959215	36016	90082938	109721	71677120	72455	118991161	117831
	Ebola	4733413	51117	946000	53465	3732476	70736	4122922	100238	4687630	100355	3732476	70736
	Dengue	2679746	163142	535989	57918	2134755	162571	2344694	99695	2629120	157280	2344694	99695
	Influenza	580108	39489	1752528	30935	462251	21020	508334	21713	15101	575971	15101	575971
	All	30173426	466701	70541899	523579	20745663	569846	106117546	607703	88970267	622408	135044728	578236
15	SARS-CoV-1	7374678	133005	2652762	211394	2652762	211394	2652739	211383	2652739	211383	2652739	211383
	MERS	7495710	49814	1224682	40764	5809898	106916	6412005	107189	7315184	107441	7315184	107441
	SARS-CoV-2	7326267	57890	63484669	189982	5964143	37021	90156005	123153	71735232	79346	119088222	132184
	Ebola	4737182	54589	946752	55755	3735616	75678	4126489	106635	4691704	106770	3735616	75678
	Dengue	2680123	185342	536022	63800	2135271	184450	2345444	111850	2629444	177592	2345444	111850
	Influenza	579634	44695	1751021	34958	461851	23061	507894	23904	575480	16471	575480	16471
	All	30193594	518372	70595908	581774	20759541	630327	106200576	673741	89599783	689040	135712685	642812

The main advantage of COVID-DeepPredictor is that it uses *k*-mer technique which is an alignment-free technique. Most analysis based works attempted so far have used alignment based techniques. Although, they are highly successful in detecting similarities in sequences of viruses, they take a lot of computational time. Also, alignment based techniques have the underlying constraint of homologous sequences which may not be the case every time. To mitigate these problems of alignment based techniques, alignment-free techniques (Kari et al., 2015) can be used. Alignment-free techniques are meant to be fast and can work with a large number of sequences. To prove the advantage of COVID-DeepPredictor over BLASTN^4^, which is an alignment-based technique, Table 4 is reported where different input sequences of size 50, 100, 200, 300, and 400 of SARS-CoV-2 are taken. For 50 sequences, BLASTN takes 1 h 15 min to align the sequences and to produce the subsequent results. Thereafter, such results are further required to be analyzed by machine intelligence technique to predict the virus class which takes some additional time as well. On the contrary, COVID-DeepPredictor successfully completes the job of training and testing, which involves prediction, in just 1.26 min. Similar results are also seen for the other varying sequences as well. Thus, we can conclude that an alignment-free technique is significantly faster than an alignment based technique.

**Table 4:** Runtime comparison of COVID-DeepPredictor and BLASTN.

	**Alignment-free technique**	**Alignment-based technique**
**Number of sequences of SARS-CoV-2**	**COVID-DeepPredictor (*****k*****=3) [Training and Testing (in min)]**	**BLASTN**
50	1.26	1 h 15 min
100	1.27	2 h 40 min
200	1.28	4 h 30 min
300	1.29	6 h 35 min
400	1.31	9 h 10 min

## 5. Conclusion

In the current scenario of global pandemic, it has become very important to predict SARS-CoV-2 as early as possible as both the affected and the number of death cases are increasing exponentially everyday. However, polymorphic nature of SARS-CoV-2 allows it to adapt and sustain in different kinds of environment which makes SARS-CoV-2 very hard to predict. In such scenarios, the proposed COVID-DeepPredictor can be very useful for predicting SARS-CoV-2 and other kinds of pathogenic viruses based on their genomic information very quickly as it uses an alignment-free technique. The results for COVID-DeepPredictor are highly encouraging as it shows prediction accuracy in the range of 99.51 to 99.94% for test datasets. Human health being the main concern of this work, the code for COVID-DeepPredictor along with the pre-trained model are also provided so that the scientific community can reap as much benefit as possible from it. Apart from SARS-CoV-2, COVID-DeepPredictor can also be used by pathogen laboratories to recognize the other five pathogenic viruses (SARS-CoV-1, MERS-CoV, Ebola, Dengue, and Influenza) very easily^4^ and accurately from a given genomic sequence. To achieve good performance, data preprocessing and the experiments are carried out on real-life datasets. Moreover, comparisons with popular existing prediction methods based on Linear Discriminant Analysis, Random Forests, and Gradient Boosting Method are also performed to show the superiority of COVID-DeepPredictor. Additionally, accuracy and runtime of COVID-DeepPredictor are taken together to determine the value of *k* in *k*-mer, comparison among *k* values in *k*-mer, Bag-of-Descriptors (BoDs) and Bag-of-Unique-Descriptors (BoUDs) is considered along with a comparative study between COVID-DeepPredictor and Nucleotide BLAST.

## Data Availability Statement

The original contributions presented in the study are included in the article/Supplementary Material, further inquiries can be directed to the corresponding author/s.

## Author Contributions

IS designed the research. IS, NG, DM, AS, and DP analyzed data and wrote the manuscript. NG performed the experiments and collected results. All authors reviewed and approved the final version of the manuscript.

## Conflict of Interest

AS was employed by company Cognizant Technology Solutions. The remaining authors declare that the research was conducted in the absence of any commercial or financial relationships that could be construed as a potential conflict of interest.

## Footnotes

^1^https://www.ncbi.nlm.nih.gov/genome/viruses

^2^https://gisaid.org/CoV2020

^3^http://www.nitttrkol.ac.in/indrajit/projects/COVID-DeepPredictor/

^4^https://blast.ncbi.nlm.nih.gov/Blast.cgi?PROGRAM=blastn&PAGE_TYPE=BlastSearch&BLAST_SPEC=&LINK_LOC=blasttab&LAST_PAGE=blastn

## References

[B1] Alagaili A. N. Briese T. Mishra N. Kapoor V. Sameroff S. C. de Wit E.. (2014). Middle east respiratory syndrome coronavirus infection in dromedary camels in Saudi Arabia. MBio 5:e00884–14. 10.1128/mBio.01002-14 24570370 PMC3940034

[B2] Chan J. F.-W. Yuan S. Kok K.-H. Kai-Wang K. Chu H. Yang J.. (2020). A familial cluster of pneumonia associated with the 2019 novel coronavirus indicating person-to-person transmission: a study of a family cluster. Lancet 395, 514–523. 10.1016/S0140-6736(20)30154-9 31986261 PMC7159286

[B3] Cui J. Li F. Shi Z.-L. (2019). Origin and evolution of pathogenic coronaviruses. Nat. Rev. Microbiol. 17, 181–192. 10.1038/s41579-018-0118-9 30531947 PMC7097006

[B4] Guan Y. Zheng B. He Y. Liu X. Zhuang Z. Cheung C.. (2003). Isolation and characterization of viruses related to the sars coronavirus from animals in southern China. Science 302, 276–278. 10.1126/science.1087139 12958366

[B5] Hinton G. E. Roweis S. T. (2003). “Stochastic neighbor embedding,” in Advances in Neural Information Processing Systems (Vancouver, BC), 857–864.

[B6] Hochreiter S. Schmidhuber J. (1997). Long short-term memory. Neural Comput. 9, 1735–1780. 10.1162/neco.1997.9.8.1735 9377276

[B7] Huang C. Wang Y. Li X. Ren L. Zhao J. Hu Y.. (2020). Clinical features of patients infected with 2019 novel coronavirus in Wuhan, China. Lancet 395, 497–506. 10.1016/S0140-6736(20)30183-5 31986264 PMC7159299

[B8] Jenkins G. M. Rambaut A. Pybus O. G. Holmes E. C. (2002). Rates of molecular evolution in RNA viruses: a quantitative phylogenetic analysis. J. Mol. Evol. 54, 156–165. 10.1007/s00239-001-0064-3 11821909

[B9] Jin Y. Luo C. Guo W. Xie J. Wu D. Wang R. (2019). Text classification based on conditional reflection. IEEE Access 7, 76712–76719. 10.1109/ACCESS.2019.2921976

[B10] Kari L. Hill K. A. Sayem A. S. Karamichalis R. Bryans N. Davis K.. (2015). Mapping the space of genomic signatures. PLoS ONE 10:e119815. 10.1371/journal.pone.0119815 PMC4441465 26000734

[B11] Khattak F. K. Jeblee S. Pou-Prom C. Abdalla M. Meaney C. Rudzicz F. (2019). A survey of word embeddings for clinical text. J. Biomed. Informatics 4:100057. 10.1016/j.yjbinx.2019.100057 34384583

[B12] Kim K. Kim D. Noh J. Kim M. (2018). Stable forecasting of environmental time series via long short term memory recurrent neural network. IEEE Access 6, 75216–75228. 10.1109/ACCESS.2018.2884827

[B13] Koohi-Moghadam M. Wang H. Wang Y. Yang X. Li H. Wang J.. (2019). Predicting disease-associated mutation of metal-binding sites in proteins using a deep learning approach. Nat. Mach. Intell. 1, 561–567. 10.1038/s42256-019-0119-z

[B14] Letko M. Marzi A. Munster V. (2020). Functional assessment of cell entry and receptor usage for SARS-CoV-2 and other lineage b betacoronaviruses. Nat. Microbiol. 5, 562–569. 10.1038/s41564-020-0688-y 32094589 PMC7095430

[B15] Liu J. Xia C. Yan H. Xie Z. Sun J. (2019). Hierarchical comprehensive context modeling for Chinese text classification. IEEE Access 7, 154546–154559. 10.1109/ACCESS.2019.2949175

[B16] Liu X. Wang X.-J. (2020). Potential inhibitors against 2019-nCoV coronavirus m protease from clinically approved medicines. J. Genet. Genomics. 47, 119–121. 10.1016/j.jgg.2020.02.001 32173287 PMC7128649

[B17] Lu R. Zhao X. Li J. Niu P. Yang B. Wu H.. (2020). Genomic characterisation and epidemiology of 2019 novel coronavirus: implications for virus origins and receptor binding. Lancet 395, 565–574. 10.1016/S0140-6736(20)30251-8 32007145 PMC7159086

[B18] Luque A. Carrasco A. Martín A. de las Heras A. (2019). The impact of class imbalance in classification performance metrics based on the binary confusion matrix. Pattern Recogn. 91, 216–231. 10.1016/j.patcog.2019.02.023

[B19] Manekar S. Sathe S. (2018). A benchmark study of k-mer counting methods for high-throughput sequencing. Gigascience 7:giy125. 10.1093/gigascience/giy125 30346548 PMC6280066

[B20] Meng Y. Wu P. Lu W. Liu K. Ma K. Huang L.. (2020). Sex-specific clinical characteristics and prognosis of coronavirus disease-19 infection in Wuhan, China: a retrospective study of 168 severe patients. PLoS Pathol. 16:e1008520. 10.1371/journal.ppat.1008520 32343745 PMC7209966

[B21] Ozturk T. Talo M. Yildirim E. A. Baloglu U. B. Yildirim O. Rajendra Acharya U. (2020). Automated detection of covid-19 cases using deep neural networks with x-ray images. Comput. Biol. Med. 121:103792. 10.1016/j.compbiomed.2020.103792 32568675 PMC7187882

[B22] Paraskevis D. Kostaki E. Magiorkinis G. Panayiotakopoulos G. Sourvinos G. Tsiodras S. (2020). Full-genome evolutionary analysis of the novel corona virus (2019-nCoV) rejects the hypothesis of emergence as a result of a recent recombination event. Infect. Genet. Evol. 79:104212. 10.1016/j.meegid.2020.104212 32004758 PMC7106301

[B23] Solis-Reyes S. Avino M. Poon A. Kari L. (2018). An open-source k-mer based machine learning tool for fast and accurate subtyping of HIV-1 genomes. PLoS ONE 13:e206409. 10.1371/journal.pone.0206409 30427878 PMC6235296

[B24] Su S. Wong G. Shi W. Liu J. Lai A. C. Zhou J.. (2016). Epidemiology, genetic recombination, and pathogenesis of coronaviruses. Trends Microbiol. 24, 490–502. 10.1016/j.tim.2016.03.003 27012512 PMC7125511

[B25] Tang B. Pan Z. Yin K. Khateeb A. (2019). Recent advances of deep learning in bioinformatics and computational biology. Front. Genet. 10:214. 10.3389/fgene.2019.00214 30972100 PMC6443823

[B26] Wan Y. Shang J. Graham R. Baric R. S. Li F. (2020). Receptor recognition by the novel coronavirus from Wuhan: an analysis based on decade-long structural studies of SARS coronavirus. J. Virol. 94:e00127-20. 10.1128/JVI.00127-20 31996437 PMC7081895

[B27] Weiss S. Navas-Martin S. (2005). Coronavirus pathogenesis and the emerging pathogen severe acute respiratory syndrome coronavirus. Microbiol. Mol. Biol. Rev. 4, 635–664. 10.1128/MMBR.69.4.635-664.2005 16339739 PMC1306801

[B28] Woo P. C. Lau S. K. Huang Y. Yuen K.-Y. (2009). Coronavirus diversity, phylogeny and interspecies jumping. Exp. Biol. Med. 234, 1117–1127. 10.3181/0903-MR-94 19546349

[B29] Worldometer (2021). Coronavirus Disease 2019 (COVID-19) Cases. Available online at: https://www.worldometers.info/coronavirus (accessed January 8, 2021).

[B30] Yan L. Zhang H.-T. Goncalves J. Xiao Y. Wang M. Guo Y.. (2020). An interpretable mortality prediction model for covid-19 patients. Nat. Mach. Intell. 2, 283–288. 10.1038/s42256-020-0180-7

[B31] Yan Q. Weeks D. E. Xin H. Swaroop A. Y. E. Chew E. Huang H.. (2020). Deep-learning-based prediction of late age-related macular degeneration progression. Nat. Mach. Intell. 2, 141–150. 10.1038/s42256-020-0154-9 32285025 PMC7153739

[B32] Zhang Y. Zheng J. Jiang Y. Huang G. Chen R. (2019). A text sentiment classification modeling method based on coordinated CNN-LSTM-attention model. Chinese J. Electron. 28, 120–126. 10.1049/cje.2018.11.004

[B33] Zhou P. Yang X. L. Wang X. G. Hu B. Zhang L. Zhang W.. (2020). A pneumonia outbreak associated with a new coronavirus of probable bat origin. Nature 579, 270–273. 10.1038/s41586-020-2012-7 32015507 PMC7095418

