# Supplementary material for: COVID-DeepPredictor: Recurrent Neural Network to Predict SARS-CoV-2 and Other Pathogenic Viruses
Source: Front Genet. 2021 Feb 11;12:569120. doi: 10.3389/fgene.2021.569120 (PMC7906283; doi:10.3389/fgene.2021.569120)
Supplement: Supplementary file 1 [file Data_Sheet_1.PDF]

Supplementary to "COVID-DeepPredictor: Recurrent Neural Network to Predict SARS-CoV-2 and Other Pathogenic Viruses"

Indrajit Saha[a,g,*], Nimisha Ghosh[b,g], Debasree Maity[c], Arijit Seal[d], Dariusz Plewczynski[e]

[a]*Department of Computer Science and Engineering, National Institute of Technical Teachers' Training and Research, Kolkata, India*

[b]*Department of Computer Science and Information Technology, Institute of Technical Education and Research, Siksha 'O' Anusandhan (Deemed to be University), Bhubaneswar,India*

[c]*Department of Electronics and Communication Engineering, MCKV Institute of Engineering, Howrah, India*

[d]*Cognizant Technology Solutions Pvt.Ltd, Kolkata, India*

[e]*Laboratory of Bioinformatics and Computational Genomics, Faculty of Mathematics and Information Science, Warsaw University of Technology, Warsaw, Poland*

[f]*Laboratory of Functional and Structural Genomics, Centre of New Technologies, University of Warsaw, Warsaw, Poland*

[g]*Equally contributed*

Abstract

The COVID-19 disease for Novel coronavirus (SARS-CoV-2) has turned out to be a global pandemic. The high transmission rate of this pathogenic virus demands an early prediction and proper identification for the subsequent treatment. However, polymorphic nature of this virus allows it to adapt and sustain in different kinds of environment which makes it difficult to predict. On the other hand, there are other pathogens like SARS-CoV-1, MERS-CoV, Ebola, Dengue and Influenza as well, so that a predictor is highly required to distinguish them with the use of their genomic information. To mitigate this problem, in this work COVID-DeepPredictor is proposed on the framework of deep learning to identify an unknown sequence of these pathogens. COVID-DeepPredictor uses Long Short Term Memory as Recurrent Neural Network for the underlying prediction with an alignment-free technique. In this regard, k-mer technique is applied to create Bag-of-Descriptors (BoDs) in order to generate Bag-of-Unique-Descriptors (BoUDs) as vocabulary and subsequently embedded representation is prepared for the given virus sequences. This predictor is not only validated for the dataset using $\mathcal{K}$-fold cross-validation but also for unseen test datasets of SARS-CoV-2 sequences and sequences from other viruses as well. To verify the efficacy of COVID-DeepPredictor, it has been compared with other state-of-the-art prediction techniques based on Linear Discriminant Analysis, Random Forests and Gradient Boosting Method. COVID-DeepPredictor achieves 100% prediction accuracy on validation dataset while on test datasets, the accuracy ranges from 99.51% to 99.94%. It shows superior results over other prediction techniques as well. In addition to this, accuracy and runtime of COVID-DeepPredictor are considered simultaneously to determine the value of k in k-mer, a comparative study among k values in k-mer, Bag-of-Descriptors (BoDs) and Bag-of-Unique-Descriptors (BoUDs) and a comparison between COVID-DeepPredictor and Nucleotide BLAST have also been performed. The code, training and test datasets used for COVID-DeepPredictor are available at *http://www.nitttrkol.ac.in/indrajit/projects/COVID-DeepPredictor/*

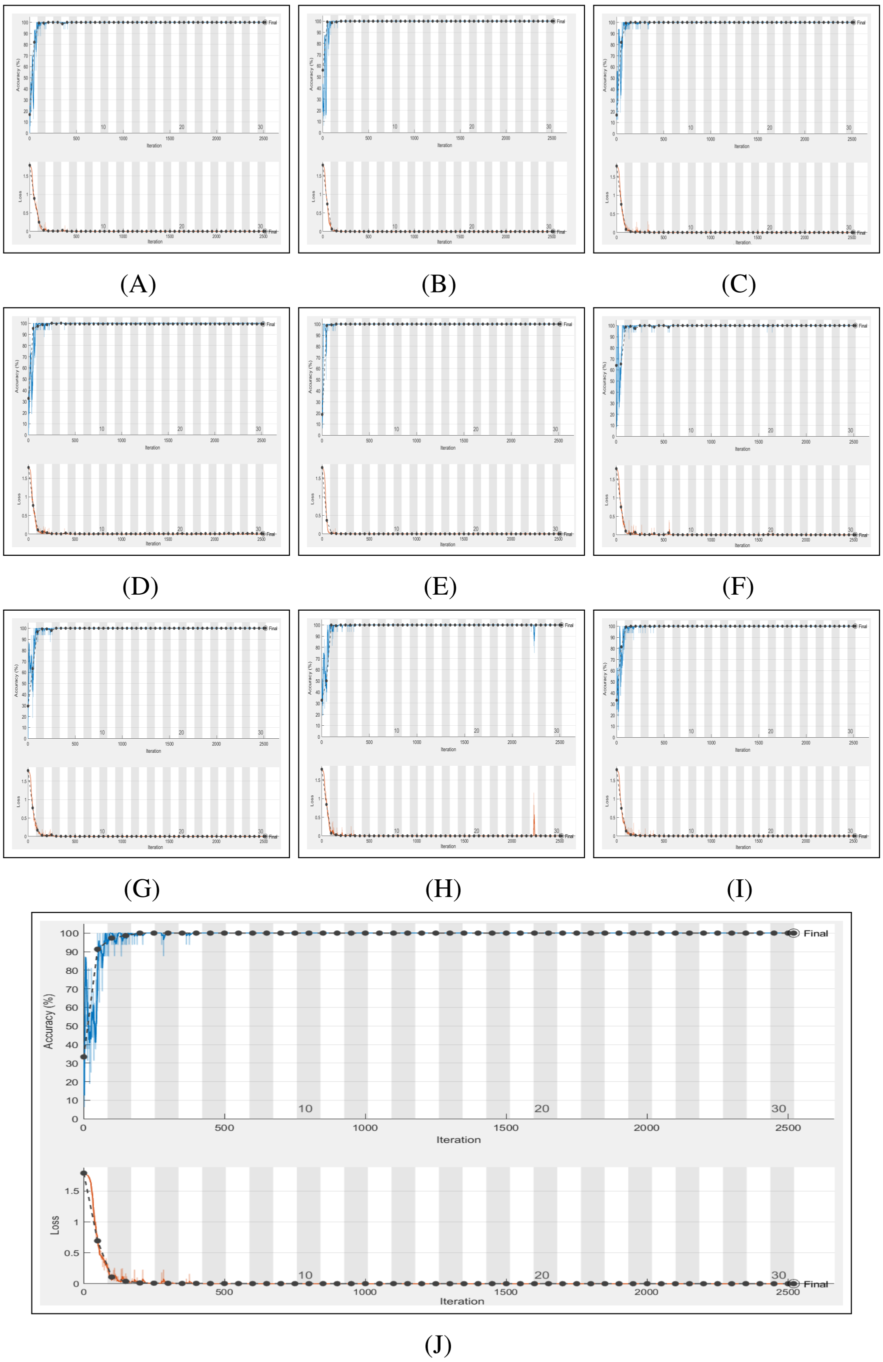

Figure S1: Convergence plots of COVID-DeepPredictor with $\mathcal{K} = 10$ in $\mathcal{K}$-fold cross-validation for k=3 in k-mer

*Corresponding author: indrajit@nitttrkol.ac.in

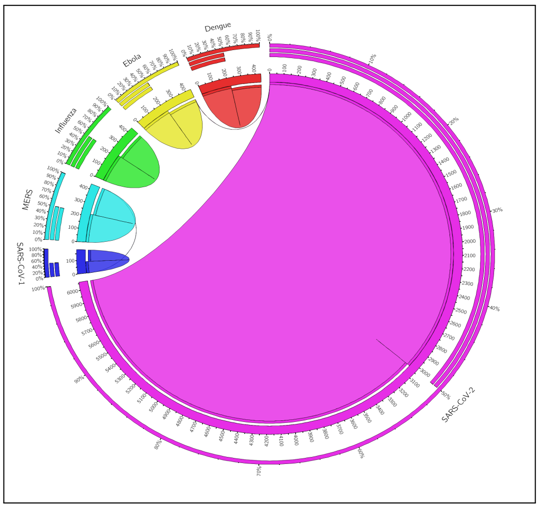

(A)

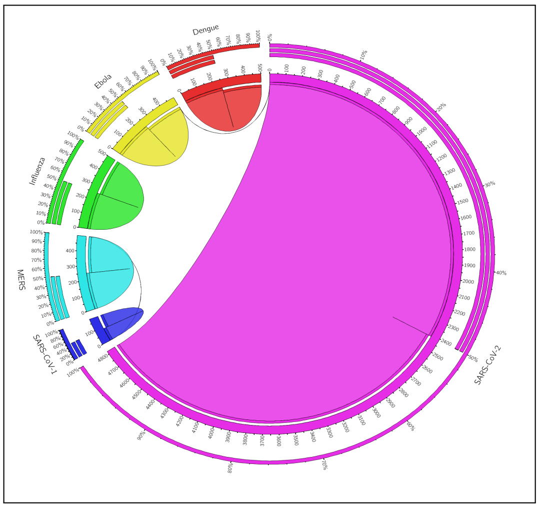

(B)

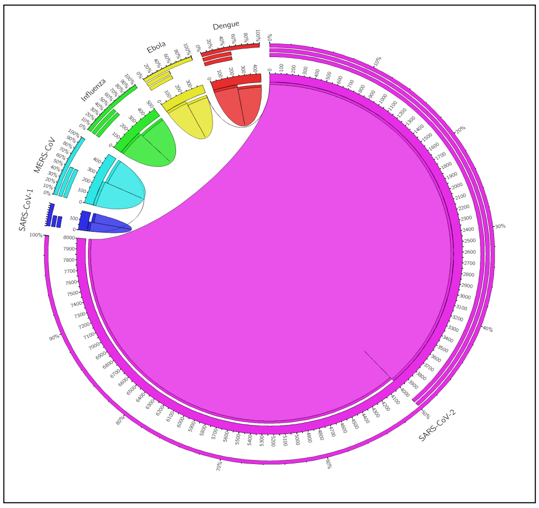

(C)

Figure S2: **Circos plot of confusion matrix for COVID-DeepPredictor for (A)** Testdata-3 (*k*=3) **(B)** Testdata-4 (*k*=3) **(C)** Testdata-5 (*k*=3).
